# Influence of Ceramsite with Assembly Unit of Sludge and Excavated Soil on the Properties of Cement Concrete

**DOI:** 10.3390/ma15093164

**Published:** 2022-04-27

**Authors:** Jiahui Wang, Shiyu Wang, Hui Wang, Zhimin He

**Affiliations:** 1 College of Civil Engineering, Nanjing Forestry University, Nanjing 210037, China; wangjiahui11@126.com; 2 School of Civil and Environmental Engineering, Ningbo University, Ningbo 315000, China; wangshiyu@sribs.com (S.W.); hezhimin@nbu.edu.cn (Z.H.); 3 Shanghai Research Institute of Building Sciences Co., Ltd., Shanghai 200032, China

**Keywords:** sludge, ceramsite, ignition loss rate, mechanical strengths, chloride ion migration coefficient, thermal conductivity

## Abstract

The application of sludge in the manufacture of ceramic material provides an outlet for waste disposal. In this study, we aimed to produce a new lightweight aggregate applications in concrete. The influence of burning temperature on the ignition loss rate, cylinder compressive strength, and the water absorption rate of ceramsite mixed with sludge and excavated soil was investigated. The slump flow, apparent density, and mechanical strength (flexural and compressive strengths) of cement concrete with ceramsite were determined. Moreover, the chloride ion permeability coefficient and the thermal conductivity were tested. Finally, scanning electron microscopy, X-ray diffraction, and thermal analysis were applied to analyze the mechanisms of the properties of ceramsite. Results show that the ignition loss rate and the burning temperature are in a quadratic relationship. The cylinder compressive strength shows a positive quadratic relationship with the burning temperature. However, the water absorption rate negatively correlates with the burning temperature. The addition of sludge can increase the ignition loss rate and cylinder compressive strength of ceramsite. Meanwhile, the effect of sludge on the water absorption rate is the opposite. Ceramsite decreases the slump flow and the apparent density of cement concrete. Cement concrete with 10% ceramsite shows the highest mechanical strength and the lowest chloride ion migration coefficient. Correction of the chloride ion migration coefficient and the content of ceramsite was performed as an exponential equation. Ceramsite exerts a negative effect on the thermal conductivity of cement concrete. Concrete with sludge ceramsite shows higher slump flow, apparent density, mechanical strength, and resistance to chloride ion penetration and thermal conductivity than concrete sludge with clay ceramsite. The mullite content of sludge ceramsite is higher than that of clay ceramsite. Additionally, sludge ceramsite exhibits a denser structure than that of clay ceramsite.

## 1. Introduction

According to statistics, engineering waste accounts for more than 75% of total construction waste due to the large-scale development of urban underground space [1,2,3]. At the same time, the discharge of industrial pollutants and waste leads an increase in the amount of polluted river sludge. By June 2020, the annual output of urban sludge in China reached more than 50 million tons, and this value is expected to reach 89.09 million tons in 2022 [4,5,6]. The accumulation of sludge in rivers deteriorates the whole water system. Using sludge as a raw material to produce industrial ceramsite may not only alleviate this issue but also bring economic benefits [7,8].

Ceramsite, invented by Hayde et al., is calcined from clay [9]. Ceramsite concrete has the advantages of high strength, low dry density, low thermal conductivity, good durability, and easy construction [10,11]. Due to these advantages, ceramsite material is used to manufacture thermal insulation concrete and sound insulation concrete [12,13]. Wang et al. [14,15] found that the bond-slip action of modified clay ceramsite with cement mortar is closely related to the temperature and water content of cement concrete. Lin et al. [16,17] reported that the addition of clay ceramsite can effectively promote resistance to fire and high temperatures in cement concrete. Additionally, Luan et al. [18,19] claimed that ceramsite can be used in the fabrication of sound absorption and sound insulation materials. With the widespread use of ceramsite in cement concrete, the consumption of clay resources will be significantly increased. 

Several scholars have made great efforts to alleviate the environmental pollution caused by river silt while simultaneously providing more resources for the production of ceramsite. Xie et al. [20] used papermaking sludge to prepare expanded ceramsite and invented a green self-insulation wall block with ceramsite as aggregate. The wall block shows an apparent density of 1020 kg/m^3^, water absorption of 14.2%, compressive strength of 5.8 MPa, and thermal conductivity of 0.39 W/(M^2^·K) [21]. Chen et al. [22] used ceramsite and blast furnace slag (GGBS) made of sludge to replace natural sand and cement as the raw material of lightweight concrete (SCRC) to prepare hollow concrete blocks, which exhibited excellent heat insulation performance and good application prospects. Zhu et al. [23] manufactured unburned ceramsite with construction waste residue, gypsum, and quicklime as a porous, sound-absorbing material for noise reduction in rail transit. This material possesses properties of good sound absorption. Light ceramsite prepared with sludge is a low-cost and lightweight organic thermal insulation material that shows high strength and non-flammability of inorganic materials and can realize the resource utilization of sludge. Although ceramsite manufactured by sludge has great potential practical value, little research has been conducted involving a systematic study of the properties (macro and micro properties) of silt ceramsite. Additionally, little attention has been paid to the comprehensive performance of silt ceramsite, including its mechanical, durability, and thermal conductive properties.

In this research, the influence of burning temperature on the physical and mechanical properties of ceramsite was investigated. The slump flow and the mechanical strengths (flexural and compressive strengths) were determined. Moreover, the water absorption rate, chloride ion permeability coefficient, and thermal conductivity were tested. Finally, microscopic experiments, including scanning electron microscopy (SEM), X-ray diffraction (XRD), and thermogravimetric (TG) analysis, were performed to analyze the mechanism of the corresponding macro properties. This research may provide guidelines for the application of silt ceramsite concrete in the future.

## 2. Experimental Methods

### 2.1. Raw Materials

Sludge was collected from a river in Huainan City, Anhui Province. The excavated soil was from subway excavation, and the clay was ordinary clay from Huainan City. 

The ceramsite in this study was produced by Anhui Taotianxia Environmental Protection Technology Co., Ltd., Huainan, China. Ordinary Portland cement with a grade of 42.5, provided by Zhucheng Yangchun Cement Co., Ltd., Zhucheng City, China, was applied to manufacture the ceramsite concrete.

### 2.2. Sample Preparation

**Figure 1:**
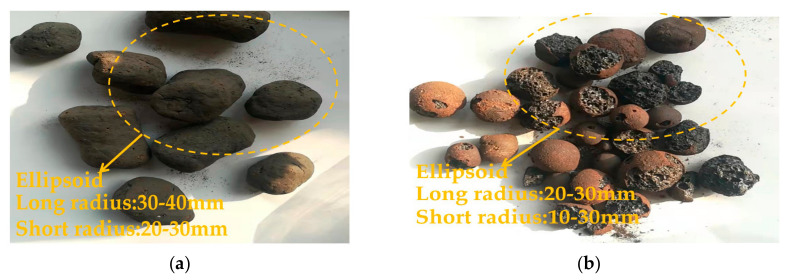
Photos of sludge ceramsite and clay ceramsite. (**a**) Sludge ceramsite. (**b**) Clay ceramsite.

The fabrication process of ceramsite can be summarized as follows. The raw materials were dried in a vacuum drying oven at (105 ± 1) °C to constant weight and then ground to power to pass through a 100 mesh standard sieve. All weighed clay or the assembly unit of subway excavation and sludge were dried in the drying oven for another two hours and then moved to a DK-98-II electronic temperature-regulating universal resistance furnace for high-temperature sintering. The furnace temperature increased from 20 °C to 1000 °C at a rate of 30 °C/min. Once the final temperature was reached, it was kept constant for 40 min. After cooling, lightweight ceramsite aggregate was obtained. The manufactured sludge and clay ceramsites were both in ellipsoids shapes. Specifically, the corresponding long and short radii of sludge ceramsites were 30~40 and 20~30 mm, respectively. The corresponding long and short radii of clay ceramsites were 20~30 and 10~30 mm, respectively. The sludge ceramsites and clay ceramsites are shown in Figure 1. 

Cement concrete with ceramsite was manufactured with the mixing proportions shown in Table 1. Ceramsite cement concrete was manufactured as follows. The powdery materials, including cement, ceramsite sand, and ceramsite, were mixed in a UJZ-15 mortar mixer for two minutes. Then, the hybrid solution of water and water-reducing agent was added and mixed for another two minutes. After that, the fresh cement concrete was poured to form the specimens with sizes of 100 mm × 100 mm × 100 mm, 100 mm × 100 mm × 400 mm, and Φ100 mm × 50 mm. The ceramsite cement concrete was manufactured in accordance with Chinese standard JGJ/T 12-2019 [24].

### 2.3. Measurement Methods

#### 2.3.1. Basic Properties of Ceramsite

Apparent density and water absorption of lightweight ceramsite aggregate can be measured according to GB/T 17431.2-2010 [25]. The apparent density measurement was conducted as follows. The sample was sieved with a standard 2.36 mm sieve. Then, the residue was dried in an electric thermostatic drying oven to a constant mass (*m*_0_). The dried samples (300~500 g) were weighed and immersed in a pycnometer for one hour (the floating object was pressed into the water with a metal rod with a disc at the end), and then the volume (*V*_0_) was obtained. The apparent density (*ρ*_0_) can be calculated according to the following Equation.

(1) $$\rho_{0}=\frac{m_{0}}{V_{0}}$$

The water absorption rate (*ω*_α_) of ceramsite was determined as following. The ceramsite (300~500 g) was dried to a constant weight in a DZF intelligent vacuum drying oven (*m*_1_). Then, the mass of ceramsite was immersed in the water and weighed until the mass (*m*_t_) was unchanged. The water absorption rate was calculated with Equation (2).

(2) $$\omega_{\alpha }=\frac{m_{t}-m_{1}}{m_{1}}$$

The cylindrical compressive strength can be determined following three steps. The light weight bearing cylinder was used to measure cylindrical compressive strength. A sample with a particle size of 10–20 mm, in which the volume content of 10–15 mm particle size was 50–70%, was selected to determine cylindrical compressive strength. The sample was filled with the pressure cylinder (with cylinder bottom) and vibrated for compaction. After that, a load with a speed of 0.3~0.5 kN/s was applied to the cylindrical specimen. Once the indentation depth reached 20 mm, recorded the pressing (*p*_1_).

The cylindrical compressive strength (*f*_α_) can be calculated by Equation (3), where *A* is the pressure-bearing area.

(3) $$f_{\alpha }=\frac{p_{1}}{A}$$

#### 2.3.2. Workability of Cement Concrete 

The inner wall and bottom plate of the slump cylinder were wet by water. Then, the bottom plate was placed on the center of a solid horizontal plane. After that, the treads on both sides were stepped by feet, and the slump cylinder remained in a fixed position during loading. The fresh cement concrete was put into the barrel with three steps, and each layer was tamped 25 times with a tamping rod. Finally, the slump cylinder was quickly removed following the vertical direction, and the slump was determined. This measurement follows GB50164-2011 [26]. 

#### 2.3.3. Mechanical Strengths of Cement Concrete

Specimens with sizes of 100 mm × 100 mm × 100 mm and 100 mm × 100 mm × 400 mm were selected for measurement of compressive and flexural strengths, respectively. The loading rates of cement concrete’s flexural and compressive strengths were 0.08~0.1 MPa and 0.3~0.8 Mpa, respectively. 

#### 2.3.4. Chloride Ion Permeability and Thermal Conductivity

Before measuring chloride ion permeability, all Φ100 mm × 50 mm specimens were moved to an automatic salt- and water-filling machine provided by Hebei AoXiang Instrument Equipment Co., Ltd., Dongguan, China, for water saturation. After that, an SX-RCM concrete chloride permeability tester (Cangzhou Kexing Instrument Equipment Co., Ltd., Cangzhou, China) was used to determine the chloride ion permeability coefficient. The chloride ion permeability coefficient of cement concrete was determined according to GB/T500820-2009 [27]. A Germany Netzsch dil402 thermal expansion instrument was used to determine thermal conductivity.

#### 2.3.5. Microscopic Characterization

Scanning electron microscopy (SEM), X-ray diffraction (XRD) and thermogravimetric (TG) analysis were performed as follows. A soybean-sized sample of ceramsite was taken out from the inner specimens. Meanwhile, part of the sample was ground into powder. The selected samples were immersed in absolute ethanol for four days to prevent the hydration of cement. After that, all samples were dried in a vacuum drying oven at 60 °C for four days. The dried samples were sprayed with a gold film before measurement. After that, a SEM (Hitachi Limited, Tokyo, Japan) experiment was carried out. The ground powder was moved to a D8 ADVANCE X-ray diffractometer (Bruker Corp., Tokyo, Japan) and a TGA 4000 thermogravimetric analyzer provided by Perkin Elmer Instrument Co., Ltd., New York, NY, USA for XRD and TG measurements, respectively.

## 3. Results and Discussions

### 3.1. Basic Properties of Ceramsite

**Figure 2:**
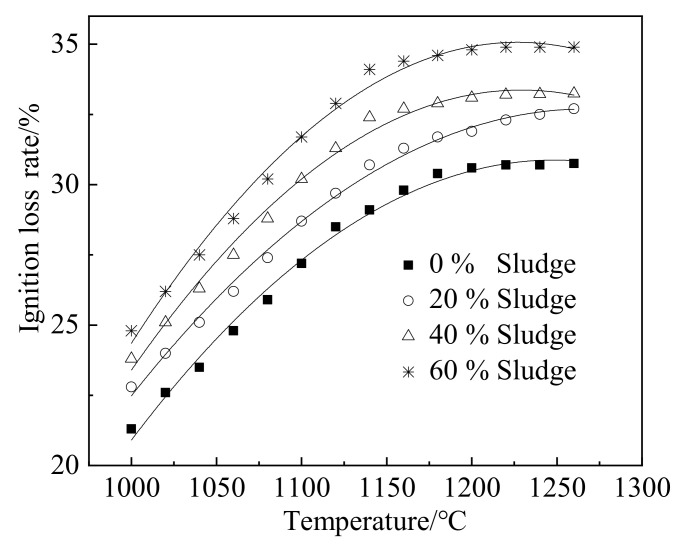
Ignition loss rate of ceramsite.

Figure 2 shows the ignition loss rate (*ILR*) of ceramsite. It can be observed from Figure 2 that the ignition loss rate of ceramsite increases obviously with the temperature rising from 1000 °C to 1180 °C. However, when the temperature ranged from 1180 °C to 1280 °C, the ignition loss rate of ceramsite tended to be stable. This may be caused by the reaction acceleration of substances in clay and silt with increasing temperature [28]. With the rising temperature, the ignition loss rate increases. More and more glassy structures are formed, leading to the structure of the ceramsite surface gradually changing from loose and porous to dense and hard. Thus, the ignition loss rates of ceramsite tends to become stable when the temperature is higher than 1180 °C [29]. Moreover, as depicted in Figure 2, an increasing dosage of sludge leads to an increased ignition loss rate of ceramsite. This is attributed to the high content of organic substance in sludge, which is decomposed during the heating process, thus increasing the ignition loss rate of ceramsite. Table 2 shows the fitted results of the relationship between ignition loss rate and temperature. A quadratic relationship is found. The high R-squared value of 0.99 indicates the accuracy of the fitting equations.

**Figure 3:**
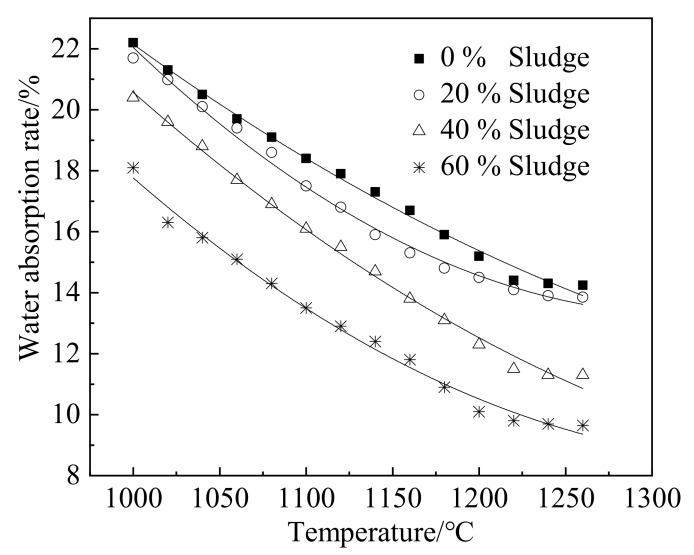
Water absorption rate of ceramsite.

Figure 3 shows the water absorption rate (*WAR*) of ceramsite, which decreases with the increasing temperature. Moreover, increasing sludge content results in a decline in the water absorption rate. This can be attributed to the fact that the rising temperature promotes the reaction of substances, thus improving the compactness of ceramsite and decreasing the water absorption rate [28]. The increasing temperature increases the glassy structures and makes water absorption stable [30]. Additionally, the increasing sludge leads to a decrease in the water absorption rate of ceramsite. This can be attributed to the fact that the active substance reacts with silica in sludge, which may form dense substances and thus reduce the water absorption rate of ceramsite [31,32]. Table 3 shows the fitting results of the relationship between water absorption rate and temperature. 

**Figure 4:**
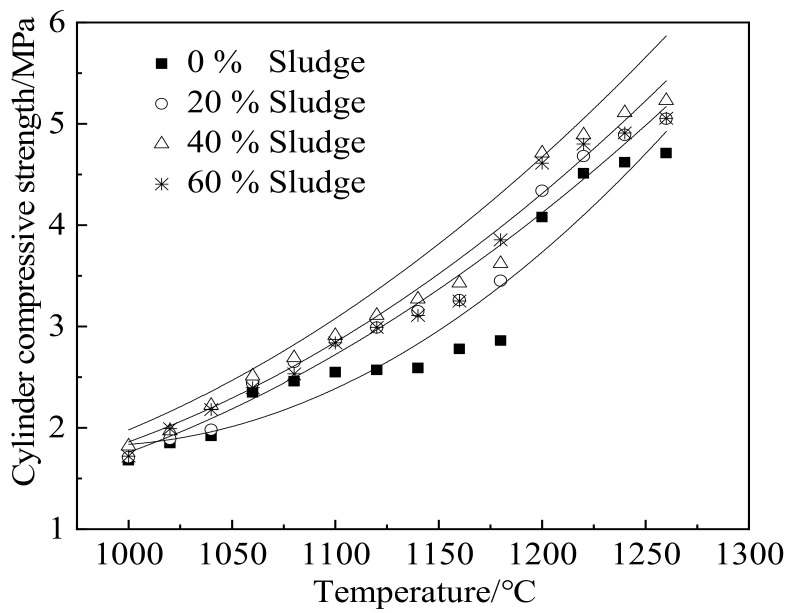
Cylinder compressive strength of ceramsite containing different contents of sludge.

Figure 4 shows the cylinder compressive strength (*f*_c_) of ceramsite, which increases with increasing temperature. Increasing sludge results in the improvement of cylinder compressive strength. This can be attributed to the fact that the rising temperature increases the compactness of ceramsite and thus improves the cylinder compressive strength of ceramsite [33,34]. Additionally, the increasing dosage of sludge from 0% to 40% increases the cylinder compressive strength of ceramsite. The overall calcium oxide content is relatively low at a low proportion of sludge, increasing the sintering temperature [35,36]. Therefore, the average pore diameter in the ceramsite increases, thus reducing the skeleton composition in the sintered ceramsite. Consequently, the cylindrical compressive strength decreases. With the increasing proportion of sludge, the inner flux or flux substances of ceramsite increase. This may lower the sintering temperature, which reduces the average pore size of ceramsite and promotes cylindrical compressive strength. However, when the sludge content is higher than 50%, the cylindrical compressive strength of ceramsite decreases with increasing dosage of sludge. This can be attributed to heavy metals and other harmful substances inducing more sintering defects in ceramsite and thus reducing the corresponding cylinder compressive strength [37,38]. Table 4 shows the fitting results of water absorption rate as a function of temperature.

### 3.2. Working and Physical Properties of Ceramsite Cement Concrete

**Figure 5:**
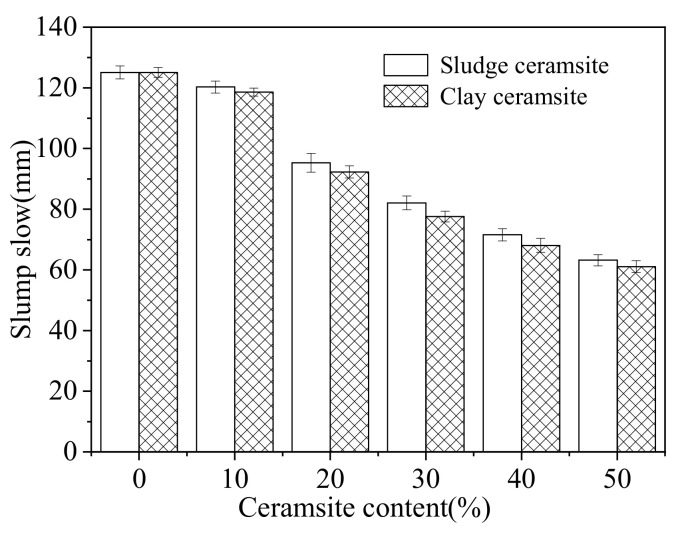
Slump flow of ceramsite cement concrete.

As obtained from the research results presented in Section 3.1, the temperature of 1180 °C is the most suitable for firing ceramsite. Moreover, the optimal raw material combination is ceramsite with 40% sludge and 60% excavated soil. Figure 5 shows the slump flow of fresh cement concrete with different dosages of ceramsite. As depicted in Figure 5, the slump flow of fresh cement concrete decreases with increasing content of ceramsite. This is because a porous material can absorb free water in the fresh cement concrete, thus reducing the slump flow [39,40]. Additionally, as illustrated in Figure 5, cement concrete with clay ceramsite shows lower slump flow than its sludge ceramsite counterpart. This is due to the lower water absorption ability of sludge ceramsite, which has lower porosity [41,42]. Therefore, the slump flow of cement concrete with sludge ceramsite is higher than that of cement concrete with clay ceramsite.

**Figure 6:**
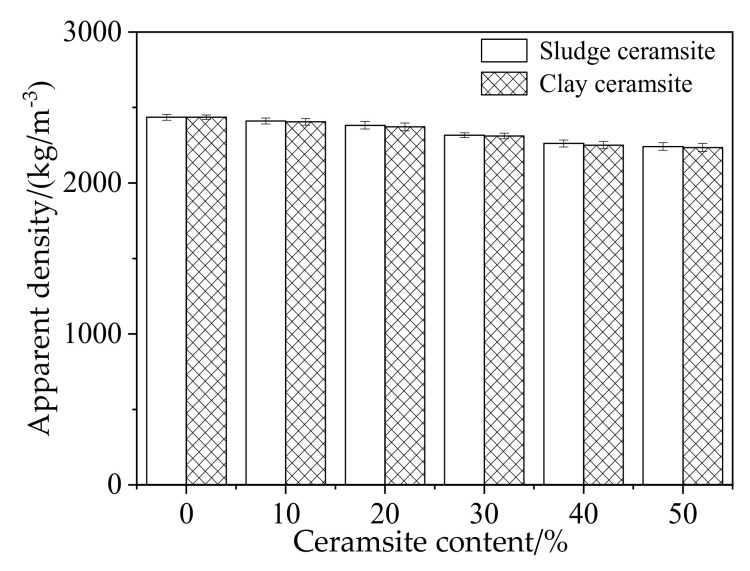
Apparent density of ceramsite cement concrete.

Figure 6 shows the apparent density of cement concrete with different dosages of ceramsite. It can be observed from Figure 6 that the apparent density of fresh cement concrete decreases with increasing dosage of ceramsite. This is because the density of porous ceramsite is lower than that of cement concrete. However, the apparent density of ceramsite cement concrete with sludge ceramsite is slightly higher than that with clay ceramsite. This is attributed to the lower density of clay ceramsite [43,44].

### 3.3. Mechanical Strengths of Ceramsite Cement Concrete

**Figure 7:**
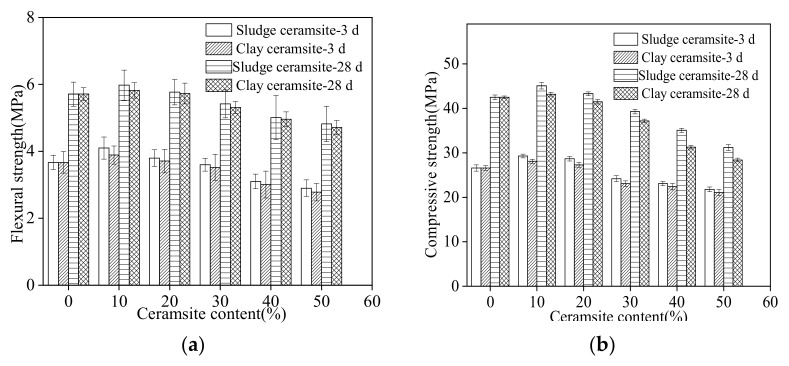
Mechanical strength of ceramsite cement concrete at different curing times. (**a**) Flexural strength. (**b**) Compressive strength.

Figure 7 shows the mechanical strengths of cement concrete with different dosages of ceramsite. The curing ages were 3 days and 28 days. The mechanical strengths of cement concrete increased with the dosage of ceramsite ranging from 0% to 10%. This was attributed to the fact that ceramsite is a porous material that can absorb free water in concrete, decreasing the water–cement ratio of cement concrete and improving mechanical strength [45,46]. However, when the content of ceramsite increases from 10% to 50%, the mechanical strength of ceramsite cement concrete decreases due to the additional defects from ceramsite. [47,48]. Cement concrete with sludge ceramsite shows higher mechanical strength than cement concrete with clay ceramsite. This can be attributed to the denser structure of sludge ceramsite [49]. As obtained from Figure 7, mechanical strength increases with increasing curing age due to the improvement of hydration degree [50].

### 3.4. Chloride Migration Coefficient

**Figure 8:**
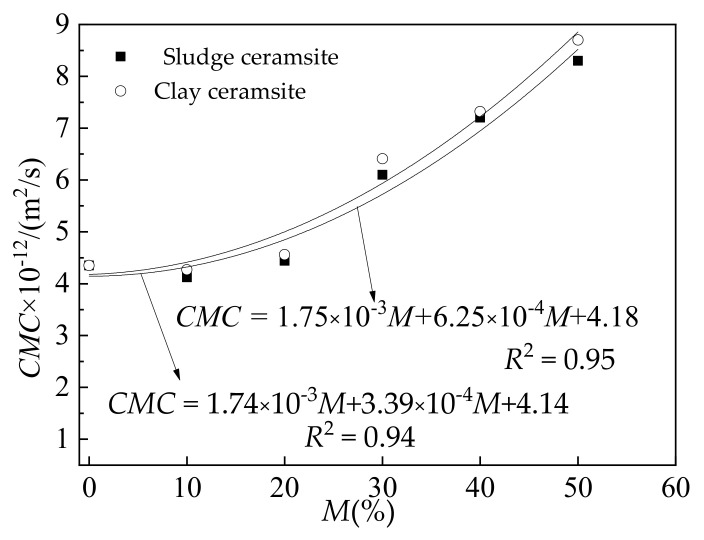
Chloride migration coefficient (CMC) of ceramsite cement concrete.

Figure 8 shows the chloride migration coefficient (CMC) of ceramsite cement concrete with different mass ratios of ceramsite by the total mass of aggregate. As obtained from Figure 8, the CMC shows a quadratic relationship with increasing dosage of ceramsite. Moreover, the CMC slightly decreases when the content of ceramsite increases from 0% to 10%. This is attributed to the fact that ceramsite can absorb free water and improve the compactness of cement concrete, thus decreasing the CMC [51,52]. However, the CMC starts to increase when the dosage of ceramsite continuously increases from 10% to 50%. This is because the ceramsite is more porous than the cement paste and ceramsite sand, which may reduce the compactness of cement concrete [53,54]. Therefore, the chloride migration coefficient of ceramsite cement concrete increases with increasing dosage of ceramsite. Furthermore, the CMC of cement concrete with sludge ceramsite is a little higher than that of cement concrete with clay ceramsite due to a denser structure [55,56].

### 3.5. Thermal Conductivity of Ceramsite

**Figure 9:**
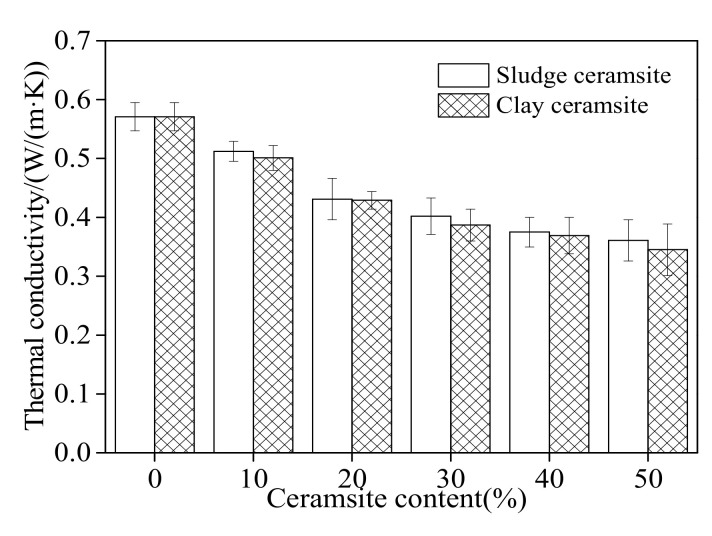
Thermal conductivity of ceramsite.

Figure 9 shows the thermal conductivity of ceramsite cement concrete, which decreases with increasing dosage of ceramsite. This can be attributed to the porous structure of ceramsite, which can reduce the thermal conductivity of concrete [57]. Cement concrete with sludge ceramsite presents higher thermal conductivity due to its better compactness.

### 3.6. Microscopic Analysis

**Figure 10:**
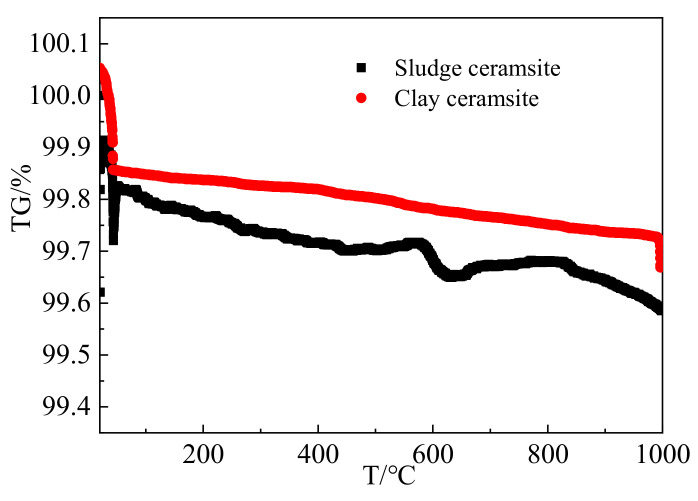
Thermogravimetric analysis curves of specimens.

Figure 10 shows the thermogravimetric analysis curves of sludge ceramsite and clay ceramsite. The temperatures in the thermogravimetric analysis curves range from 20 °C to 1000 °C. As depicted in Figure 10, the mass loss rates of sludge ceramsite and clay ceramsite samples decrease from 100% to 99.59% and 99.67%, respectively. This is attributed to the decomposition the carbonation products of alumina and silicon oxide. It can be observed from Figure 10 that the mass loss rate of sludge ceramsite is higher than that of clay ceramsite, thus confirming higher activity of sludge ceramsite than that of clay ceramsite.

**Figure 11:**
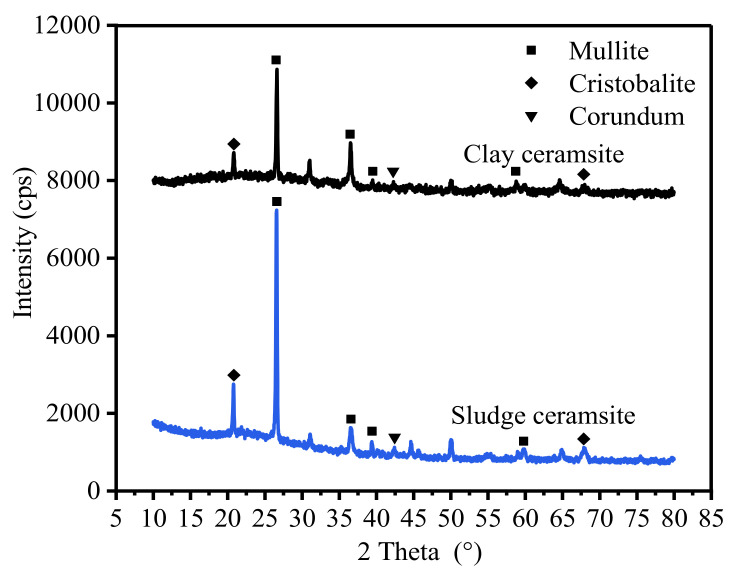
X-ray diffraction patterns of ceramsite.

The mineralogical compositions of two different ceramsites were identified through X-ray diffraction (XRD) measurements. As shown in Figure 11, the XRD patterns of both ceramsites exhibit sharp peaks, indicating the existence of crystalline phases. These peaks can be attributed to the diffraction of mullite, cristobalite, and corundum. The most intense peaks in both XRD patterns are locates at around 27° are belonging to mullite. The fact that sludge ceramsite possesses a higher peak intensity of mullite indicates that the mullite content of sludge ceramsite was higher. Therefore, sludge ceramsite shows more active properties than clay ceramsite.

**Figure 12:**
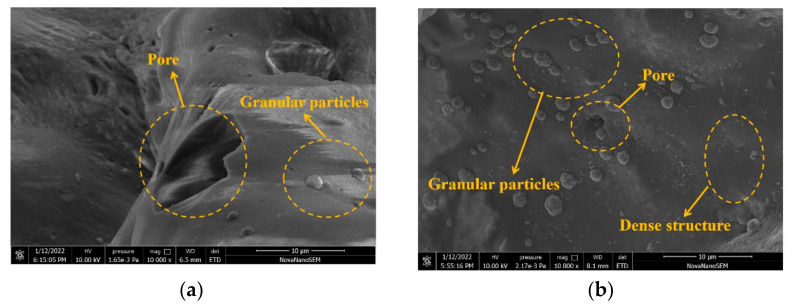
SEM micrographs of clay ceramsite and sludge ceramsite. (**a**) Clay ceramsite. (**b**) Sludge ceramsite.

The microstructure of two ceramsites were analyzed using scanning electron microscopy (SEM). As shown in Figure 12a, the clay ceramsite exhibits a rough surface embedded with small particles and pores. However, for the sludge ceramsite shown in Figure 12b, the microstructure becomes much flatter, with fewer small pores. SEM observations indicate that sludge ceramsite has a more compact and uniform microstructure than clay ceramsite. It can be observed from Figure 12 that the cement concrete with clay ceramsite has a higher pore volume ratio; therefore, the mechanical strength and thermal conductivity of clay ceramsite cement concrete are lower than those of sludge ceramsite cement concrete. These results account for the mechanism of sludge ceramsite with better mechanical strength and higher thermal conductivity.

## 4. Conclusions

In this study, the influences of sludge on the mechanical and physical properties of ceramsite were investigated. Moreover, the macroscopic and microscopic properties of cement concrete with clay and sludge ceramsite were studied. The conclusions can be summarized as follows.

The physical and mechanical properties of ceramsite are closely correlated with the sintering temperature. The ignition loss rate and cylinder compressive strength show positive quadratic relationships with the burning temperature. The relationship between water absorption rate and the burning temperature can be deduced as a negative quadratic function. A temperature of 1180 °C was the optimal sintering temperature for manufacturing ceramsite. The addition of sludge increases the ignition loss rate and cylinder compressive strength of ceramsite while decreasing the water absorption rate.

The addition of ceramsite reduces the slump flow and the apparent density of cement concrete. A proportion of 10% ceramsite is the optimum dosage for mechanical strength and resistance to chloride ion penetration of cement concrete. The correction of chloride ion migration coefficient and the content of ceramsite can be deduced as an exponential equation.

Ceramsite has a negative effect on the thermal conductivity of cement concrete. Concrete with sludge ceramsite achieves a higher slump flow, apparent density, mechanical strengths, resistance to chloride ion migration coefficient, and thermal conductivity than that with clay ceramsite. The mullite content of sludge ceramsite is higher than that of clay ceramsite. Sludge ceramsite exhibited a denser structure than that of clay ceramsite.

## Figures and Tables

**Table 1:** Mix proportions of ceramsite per one cubic meter (kg).

Cement	Water	Ceramsite Sand	Ceramsite	Water-Reducing Agent
500	200	1000	0	0.5
500	200	900	100	0.5
500	200	800	200	0.5
500	200	700	300	0.5
500	200	600	400	0.5
500	200	500	500	0.5

**Table 2:** Fitting results of the relationship between ignition loss rate and temperature.

Equation	Sludge Content/%	*a*	*b*	*c*	*R* ^2^
$ILR=aT^{2}+bT+c$	0	−1.61 × 10^−4^	0.40	−220.62	0.99
	20	−1.43 × 10^−4^	0.36	−196.55	0.99
	40	−1.90 × 10^−4^	0.47	−253.62	0.99
	60	−2.08 × 10^−4^	0.51	−278.39	0.99

**Table 3:** Fitting results of the relationship between water absorption rate and temperature.

Equation	Sludge Content/%	*a*	*b*	*c*	*R* ^2^
$WAR=aT^{2}+bT+c$	0	3.56 × 10^−5^	−0.11	98.73	0.99
	20	8.36 × 10^−5^	−0.22	159.81	0.99
	40	4.82 × 10^−5^	−0.15	118.70	0.99
	60	6.53 × 10^−5^	−0.18	132.40	0.99

**Table 4:** Fitting results of water absorption rate as a function of temperature.

Equation	Sludge Content/%	*a*	*b*	*R* ^2^
$f_{c}=a\left(1-e^{-bT}\right)$	0	−2.51 × 10^−2^	−4.18 × 10^−3^	0.93
	20	−3.13 × 10^−2^	−4.07 × 10^−3^	0.98
	40	−3.31 × 10^−2^	−4.06 × 10^−3^	0.97
	60	−3.14 × 10^−2^	−4.08 × 10^−3^	0.97

## Data Availability

The data used to support the findings of this study are available from the corresponding author upon request.
